# Vil-Cre specific Schlafen 3 knockout mice exhibit sex-specific differences in intestinal differentiation markers and Schlafen family members expression levels

**DOI:** 10.1371/journal.pone.0259195

**Published:** 2021-10-28

**Authors:** Emilie E. Vomhof-DeKrey, Allie D. Stover, Mary Labuhn, Marcus R. Osman, Marc D. Basson

**Affiliations:** 1 Department of Surgery, School of Medicine and the Health Sciences, University of North Dakota, Grand Forks, ND, United States of America; 2 Department of Biomedical Sciences, School of Medicine and the Health Sciences, University of North Dakota, Grand Forks, ND, United States of America; 3 Department of Pathology, School of Medicine and the Health Sciences, University of North Dakota, Grand Forks, ND, United States of America; Wayne State University, UNITED STATES

## Abstract

The intestinal epithelium requires self-renewal and differentiation in order to function and adapt to pathological diseases such as inflammatory bowel disease, short gut syndrome, and ulcers. The rodent Slfn3 protein and the human Slfn12 analog are known to regulate intestinal epithelial differentiation. Previous work utilizing a pan-Slfn3 knockout (KO) mouse model revealed sex-dependent gene expression disturbances in intestinal differentiation markers, metabolic pathways, Slfn family member mRNA expression, adaptive immune cell proliferation/functioning genes, and phenotypically less weight gain and sex-dependent changes in villus length and crypt depth. We have now created a Vil-Cre specific Slfn3KO (VC-Slfn3KO) mouse to further evaluate its role in intestinal differentiation. There were increases in Slfn1, Slfn2, Slfn4, and Slfn8 and decreases in Slfn5 and Slfn9 mRNA expression that were intestinal region and sex-specific. Differentiation markers, sucrase isomaltase (SI), villin 1, and dipeptidyl peptidase 4 and glucose transporters, glucose transporter 1 (Glut1), Glut2, and sodium glucose transporter 1 (SGLT1), were increased in expression in VC-Slfn3KO mice based on intestinal region and were also highly female sex-biased, except for SI in the ileum was also increased for male VC-Slfn3KO mice and SGLT1 was decreased for both sexes. Overall, the variations that we observed in these VC-Slfn3KO mice indicate a complex regulation of intestinal gene expression that is sex-dependent.

## Introduction

The murine Schlafen protein family is organized into three groups based on their molecular mass and structure. Slfn1 and -2 are the shortest and are in Group I. Slfn3 and -4 are in Group II and are intermediate in mass. The longest Slfns are in Group III and included Slfn5, -8, -9, and -14 [1, 2]. All of the Slfns share a specific slfn box domain that is next to a divergent AAA domain [2, 3]. However, a SWADL (Ser-Trp-Ala-Asp-Leu) domain appears only in the Group II and III Slfn family members. Finally, only Group III Slfns contain a C-terminal nuclear targeting sequence (RKRRR) that is homologous to DNA/RNA helicase superfamily I, and so Slfns in Groups I and II are cytosolic proteins since they do not contain this C-terminal nuclear targeting sequence [2–6]. The human Slfn proteins include SLFN5, SLFN11, SLFN12, SLFN13, and SLFN14 [7]. SLFN12 is the only protein that remains in the cytoplasm and is most closely homologous to murine Slfn3 and Slfn4 from Group II [7]. The most prominent role of this gene family is their control of cell differentiation, cell growth, and cell cycle progression [8]. Much work has also focused on the role of Slfn3, 4, 5, and 8 in the regulation of T cell differentiation, activation, and proliferation [2, 9, 10].

More specifically, Slfn3 is necessary for the regulation of intestinal differentiation, development, and maturation [11–16]. There is a direct correlation between the expression of Slfn3 and the expression of epithelial differentiation markers sucrase isomaltase (SI), villin1 (Vil1), dipeptidyl peptidase 4 (Dpp4), glucose transporter 2 (Glut2), and sodium glucose transporter 1 (SGLT1), which is dependent on the P-loop region in the N-terminus of Slfn3 [12, 15–17]. Previous work utilizing a pan-Slfn3 knockout (Slfn3KO) mouse demonstrated that Slfn3 affects the mRNA expression levels of Slfn family members in the intestinal mucosa, thymus, and spleen [18]. Additionally, the global loss of Slfn3 results in sex-dependent disturbances in metabolic pathways, genes related to adipogenesis and ketogenesis, and intestinal differentiation markers [16]. This was associated with less weight gain in the Slfn3KO mice in comparison to wild type (WT) mice, with the most striking decrease in female Slfn3KO mice [16]. We have further studies involving the Slfn3 human ortholog, SLFN12, where we demonstrated that SLFN12 promotes the expression of SI by binding to SerpinB12 through its ATP-binding region [19]. This binding of SLFN12 to SerpinB12 results in the stimulation of USP14 and UCHL5 deubiquitylase activity, leading to increases in Cdx2 levels. Determination of this pathway may allow for its targeting and manipulation in human enterocytic differentiation in order to treat mucosal atrophy, short gut syndrome, and possibly obesity [19].

The small intestinal mucosa is a heterogenous environment of immune cells and intestinal epithelial cells, and Slfn family members have functional roles in both cell populations. Therefore, we now have sought to specifically target Slfn3 in epithelial cells to better define its functional role in intestinal epithelial differentiation. We generated a Vil-Cre specific Slfn3 knockout mouse and evaluated changes in intestinal differentiation marker and Slfn family member expression.

## Materials and methods

### Generation and genotyping of VC-Slfn3KO mice

Animal breeding and procedures were approved by the University of North Dakota institutional animal use and care committee under protocol number 1807-7C. Mice under this protocol were euthanized by CO_2_ inhalation followed by cervical dislocation. The Slfn3^βgeo^ mice (strain ID: Slfn3^tm1a(KOMP)Wtsi^) were created at the UC Davis KOMP Repository (Davis, CA; Design ID: 111259; Project ID: CSD76961). The Slfn3^βgeo^ mice were bred with B6.129S4-*Gt(ROSA)26Sor*^tm2(FLP*)Sor^/J (Flpo) mice (Stock #012930) from Jackson Laboratories (Bar Harbor, ME). The resulting Slfn3^flox+/+^Flp^+/+^ pups were backcrossed with C57Bl/6J mice to remove the Flp transgene. These subsequent pups, Slfn3^flox+/-^ were crossed together to generate Slfn3^flox-/-^ mice. The Slfn3^flox-/-^ mice were bred with B6.Cg-Tg(Vil1-cre)1000Gum/J mice (Stock #021504) from Jackson Laboratories. These resultant mice, Slfn3^flox+/-^/ Flp^-/-^/Cre^+/-^, were bred together and then the following Slfn3^flox+/+^/ Flp^-/-^/Cre^+/-^ mice were bred with Slfn3^flox+/+^/Flp^-/-^ to ultimately generate the Slfn3^flox+/+^/ Flp^-/-^/Cre^-/-^ Control (Ctrl) mice and the Slfn3^flox+/+^/ Flp^-/-^/Cre^+/-^ VC-Slfn3KO mice (S1 Fig).

Genotyping protocols included isolating DNA from 2-4mm tails using the DNeasy Blood and Tissue kit and the Qiacube form Qiagen (Valencia, CA). The following PCR mix was used for genotyping PCR reactions of the Slfn3^βgeo^ mice: 25 μl total reaction = 1.3 M Betain (Sigma-Aldrich), 0.325 μl DMSO, 1.75 mM MgCl_2_, 1x Buffer without MgCl_2_, 0.2 μM forward primer, 0.2 μM reverse primer, 0.2 mM dNTPs, 0.04 U/μl AmpliTaq DNA polymerase (ThermoFisher Scientific, Waltham, MA), 2 μl DNA, and water. There are three primer pair combinations in separate genotyping PCRs utilized to genotype the Slfn3^βgeo^ mice. The first was the LoxF reaction utilizing the loxF and Slfn3-R primers. The second was the NeoF reaction utilizing the neoF and Slfn3-ttR primers. The third was the WT reaction utilizing the Slfn3-F and Slfn3-ttR primers (Table 1). PCR parameters were as follows: denaturation, 94C for 5 min; 1^st^ annealing and extension, 94C for 15 sec, 65C for 30 sec, 72C for 40 sec, 10 cycles with 1C decrease per cycle; 2^nd^ annealing and extension, 94C for 15 sec, 55C for 30 sec, 72C for 40 sec, 30 cycles; final extension, 72C for 5 min; hold at 4C. PCR reactions were analyzed on a 1.5% agarose gel stained with ethidium bromide (S2A Fig). The Slfn3^flox+/+^/Flp^+/+^ mice were genotyped with the LoxF, NeoF, and WT PCR reactions above and also with the following PCR reaction from Jackson Laboratories, Flp reaction: 12 μl total reaction = 2 mM MgCl_2_, 1x Kapa 2G HS Buffer without MgCl_2_, 0.5 μM of each primer: ROSA8052, ROSA8545, and ROSA8546 primer (Table 1), 0.2 mM dNTPs, 0.05 μl of 2.5 U/μl Kapa 2G HS Taq polymerase (Kapa Biosystems, Wilmington, MA), 2 μl DNA, and water (protocol and primer sequences from Jackson Laboratories). PCR parameters were as follows: denaturation, 94C for 2 min; 1^st^ annealing and extension, 94C for 20 sec, 65C for 15 sec, 68C for 10 sec, 10 cycles with 0.5C decrease per cycle; 2^nd^ annealing and extension, 94C for 15 sec, 60C for 15 sec, 72C for 10 sec, 28 cycles; final extension, 72C for 2 min; hold at 4C. PCR reactions were analyzed on a 1.5% agarose gel stained with ethidium bromide (S2B Fig). The Slfn3^flox+/+^/ Flp^-/-^/Cre^+/-^ (VC-Slfn3KO) and Slfn3^flox+/+^/ Flp^-/-^/Cre^-/-^ (Control (Ctrl) mice) were genotyped with the following LoxF and WT reactions (which are modifications to the original genotyping protocols from above). The LoxF qPCR reaction mix included 1x SybrGreen buffer (BioRad, Hercules, CA), 250nM loxF primer, 250nM Slfn3-R primer, 2 μl DNA, and water to a total volume of 20 μl. qPCR analysis was performed using the BioRad CFX976 Touch Real-Time Detection System with the following parameters: denaturation, 94C for 3 min; 1^st^ annealing and extension, 94C for 10 sec, 65C for 30 sec, 10 cycles with 1C decrease per cycle; 2^nd^ annealing and extension, 94C for 10 sec, 55C for 30 sec, 30 cycles, and then a melt curve was run. The WT PCR reaction mix included 1.3 M Betain, 1x GoTaq G2 Green Master Mix (Promega, Madison, WI), 0.2 μM Slfn3-F primer, 0.2 μM Slfn3-ttR primer, 2 μl DNA, and water to a total volume of 25 μl. PCR parameters were as follows, denaturation, 95C for 2 min; annealing and extension, 95C for 15 sec, 60C for 30 sec, 72C for 45 sec, 30 cycles, final extension, 72C for 5 min; hold at 4C. PCR reaction was analyzed on a 1.5% agarose gel stained with ethidium bromide (S2B and S2C Fig). Incorporation of the Vil-Cre insert was determined from the following qPCR reaction: 1x Fast EvaGreen Master Mix (VWR, Radnor, PA), 0.5 μM Vil74 primer, 0.5 μM Vil75 primer, 0.5 μM Vil76 primer (Table 1) and water to a total reaction volume of 20 μl (protocol and primer sequences from Jackson Laboratories). qPCR analysis was performed using the BioRad CFX976 Touch Real-Time Detection System with the following parameters: denaturation, 94C for 2 min; 1^st^ annealing and extension, 94C for 20 sec, 65C for 15 sec, 10 cycles with 0.5C decrease per cycle; 2^nd^ annealing and extension, 94C for 15 sec, 60C for 30 sec, 28 cycles, and then a melt curve was run (S2D Fig).

**Table 1:** Genotyping primers.

**loxF**	5’-GAG ATG GCG CAA CGC AAT TAA TG-3’
**Slfn3-R**	5’-CTT TCC TCC CCT ACT TGC TTC TTG G-3’
**neoF**	5’-GGG ATC TCA TGC TGG AGT TCT TCG-3’
**Slfn3-ttR**	5’-GGA AAA GGT GTG TCC TTG TTG GAG G -3’
**Slfn-F**	5’-CTG AAA CAT CTT AAG CCA GGA CCC C-3’
**ROSA8052**	5’- GCG AAG AGT TTG TCC TCA ACC-3’
**ROSA8545**	5’-AAA GTC GCT CTG AGT TGT TAT-3’
**ROSA8546**	5’-GGA GCG GGA GAA ATG GAT ATG-3’
**Vil74**	5’-AGG CAA ATT TTG GTG TAC GG-3’
**Vil75**	5’-GCC TTC TCC TCT AGG CTC GT-3’
**Vil76**	5’-TAT AGG GCA GAG CTG GAG GA-3’

### Single-molecule RNA in situ hybridization

RNA in situ hybridization experiments were performed by Advanced Cell Diagnostics, Inc. (Newark, CA) using the RNAscope® technology, which has been previously described [20]. Paired double-Z oligonucleotide probe was designed against Slfn3 target RNA using custom software. The Probe Design # was NPR-0001265 and was named BA-Mm-Slfn3-2zz-st. This specific probe for Slfn3 targeted 560–1785 of NM_011409.1. FFPE tissue section samples were prepared according to manufacturer’s recommendations. Each sample was quality controlled for RNA integrity with a probe specific to the housekeeping gene PPIB. Negative control background staining was evaluated using a probe specific to the bacterial dapB gene. Brightfield/fluorescent images were acquired using a Leica-SS7225 microscope with a 40x objective.

### Histology

Ileum was collected and was fixed in 10% formalin at room temperature. After 48 hours the tissue was transferred to 70% ethanol. Thereafter, tissues were processed using the Lynx II Tissue Processor according to the manufacturer’s instructions, embedded in paraffin, sectioned, and stained with the assistance of the Histology core (Department of Biomedical Sciences, University of North Dakota). 5–8μm transverse sections of the intestines were stained with H&E for further analysis.

### RNA isolation and qPCR

Intestinal mucosa was collected from 6–17 week old male and female Ctrl and VC-Slfn3KO mice. Total RNA was isolated from intestinal duodenum, jejunum, and ileum using the RNeasy Lipid Kit and the QiaCube instrument per manufacturers’ protocols (Qiagen, Valencia, CA). Preparation of cDNA from RNA samples was performed using the SMARTScribe Reverse Transcription kit (Takara Clontech, Mountain View, CA). qPCR analysis of the cDNA samples were analyzed using the BioRad CFX96 Touch Real-Time PCR Detection System and the PrimeTime Gene Expression Master Mix from Integrated DNA Technology (IDT, Coralville, IA). RNA expression levels were ascertained from the threshold cycle (Ct) values using the 2^-ΔΔCt^ method using RPLP0 as the reference control gene. Primer/probe sets and qPCR cycle conditions were previously published [16].

### Intestinal epithelial cell isolation & flow cytometry

Intestinal cells were isolated as previously described with a few modifications [21]. Briefly, intestinal regions were opened longitudinally and cut into 0.5 cm pieces and placed in a 50 ml tube with 10ml PBS. Lamina propria cells were isolated using 4 ml of 1x Liberase TM (0.2 Wunsch unit/ml, Millipore Sigma) + 200 K/ml DNase I (Millipore Sigma) in HBSS Solution (HBSS + 10 mM HEPES + 100 units/ml penicillin, 100 ug/ml streptomycin) was added and sample was incubated at 37°C for 30 min, with 200 rpm shaking. After digestion, 4 ml of cRPMI (cRPMI: 500ml RPMI, 10% FBS, 2 mM L-glutamine, 1 mM sodium pyruvate, 1x non-essential amino acids, and 100 units/ml penicillin, 100 ug/ml streptomycin, (RPMI & FBS, Genesee Scientific, Cajon, CA; all other cRMPI components from Millipore Sigma, St. Louis, MO) were added and cells were passed over a 100 μm sieve into a new 50 ml tube. Cells were centrifuged at 500 xg for 5min, 4°C. Cells were then stained for flow cytometry analysis. Cells were pretreated with TruStain fcX (Biolegend, San Diego, CA) which blocks non-specific antibody binding to Fc receptors. Extracellular staining of anti-CD3 (17A2, APC conjugated) and anti-EpCam (G8.8, APC/Cy7 conjugated) were from Biolegend. For intracellular staining of anti-sucrase isomaltase (SI) (aa144-193, FITC conjugated, LSBio, Seattle, WA) was utilized with the Foxp3/Transcription Factor Staining Set (eBioscience, San Diego, CA). Cells were acquired on a BD FACSymphony (BD, San Jose, CA) and analyzed with FlowJo software (BD).

### Statistics

Quantitative PCR was assessed by 2-way ANOVA with Uncorrected Fisher’s LSD. All other data was compared by unpaired, two-tailed t-test. We sought 95% confidence in our statistical analysis and corrected for multiple tests as necessary. Data are represented as mean ± SE.

## Results

### Generation of VC-Slfn3KO mice

**Fig 1:**
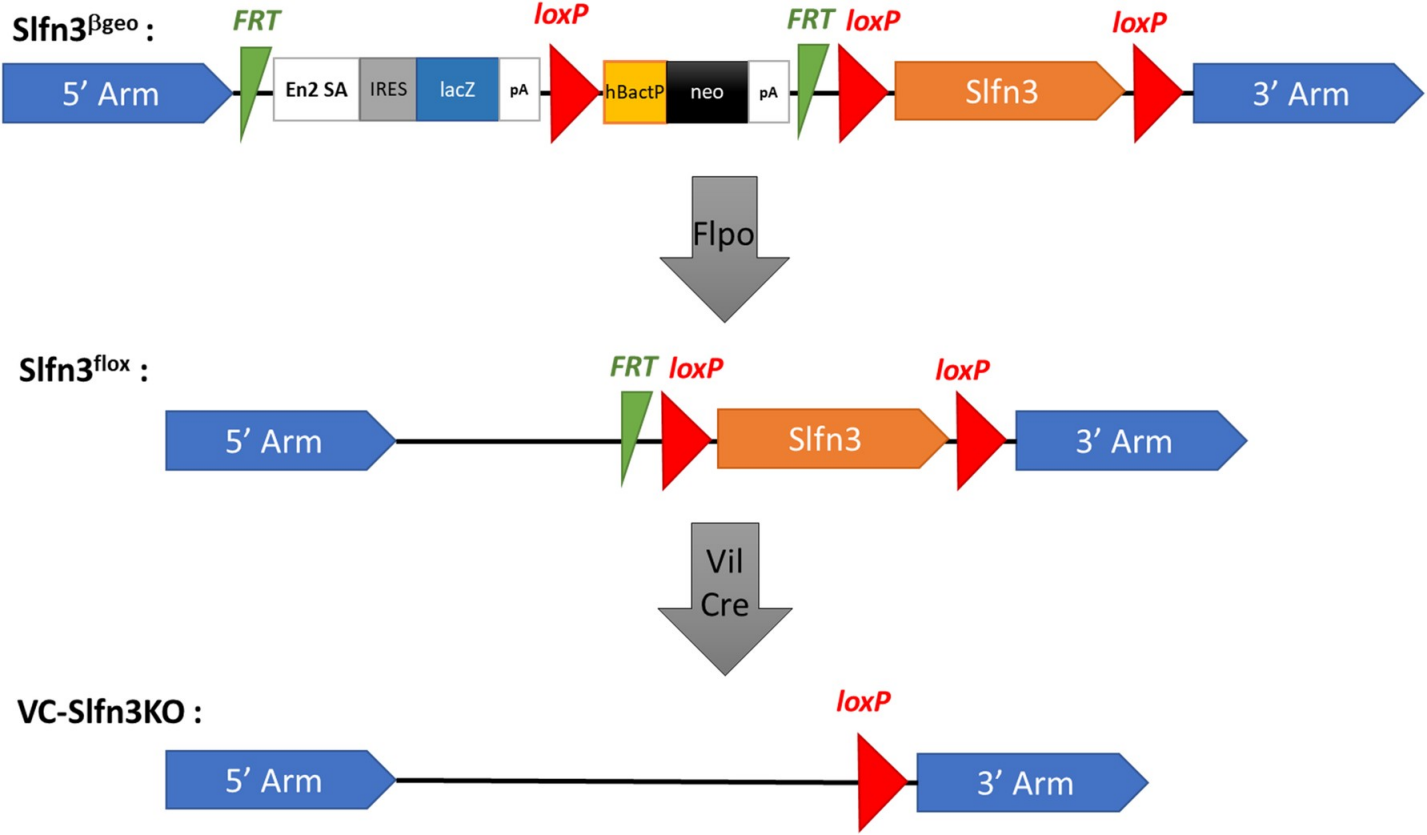
Gene targeting scheme for generating VC-Slfn3KO mice. The Slfn3^βgeo^ mice had a knockout first, *lacZ*-tagged insertion, and conditional allele of Slfn3. These mice were bred with B6.129S4-*Gt(ROSA)26Sor*^tm2(FLP*)Sor^/J (Flpo) mice, resulting in Slfn3^flox^ mice. The Slfn3^flox^ mice were then bred with B6.Cg-Tg(Vil1-cre)1000Gum/J (VilCre) mice to create VC-Slfn3KO mice.

**Fig 2:**
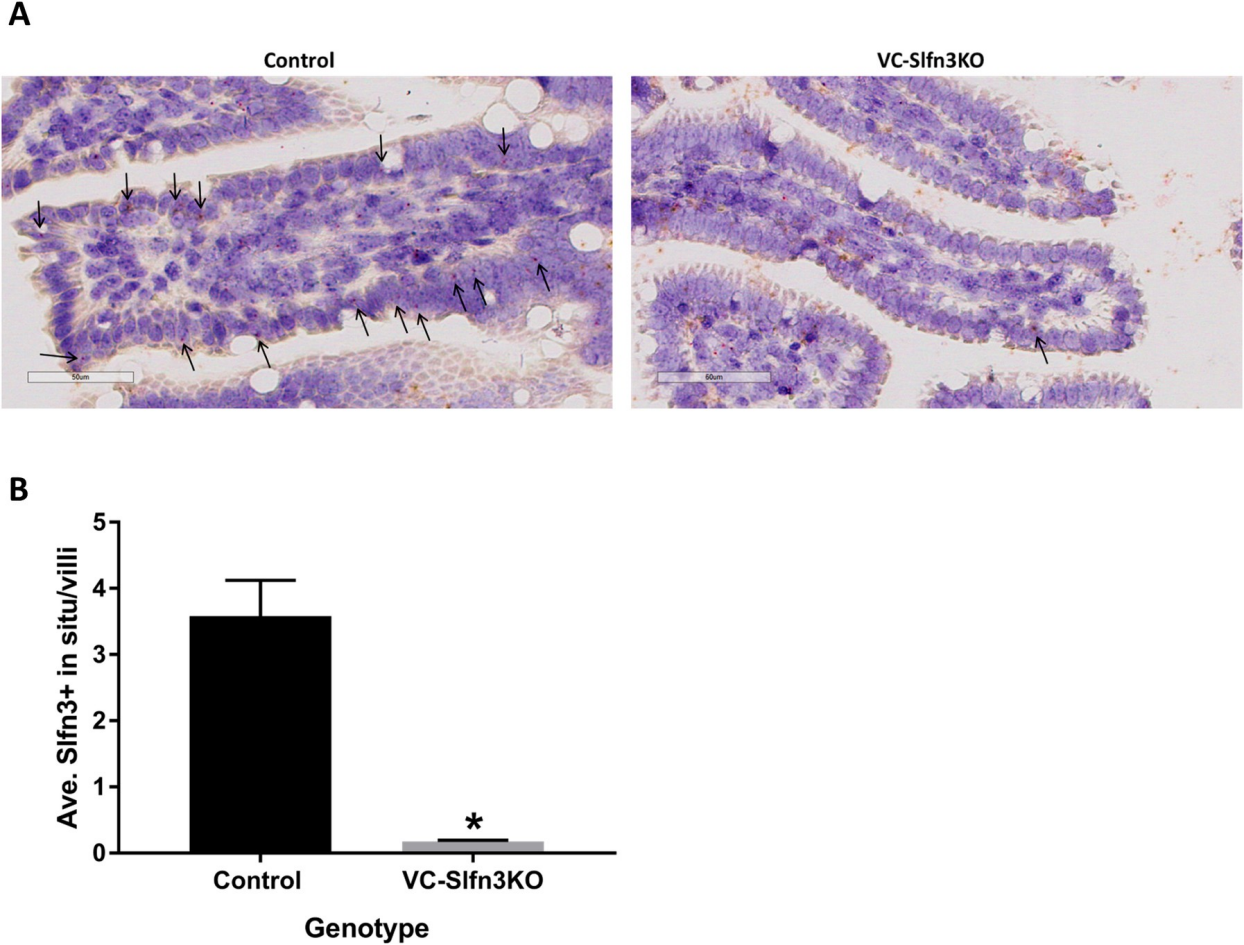
Confirmation of Slfn3 knockout in the Vil-Cre intestinal epithelial specific cells. RNA in situ hybridization experiments were performed by Advanced Cell Diagnostics, Inc. using RNAscope technology. (A) Images of Ctrl and VC-Slfn3KO villi with arrows indicating Slfn3+ intestinal epithelial cells. (B) Bar graph quantification of the average number intestinal epithelial cells that were Slfn3+ per villi (n = 3 mice/group, average of 22 villi per sample, *p ≤ 0.05).

**Fig 3:**
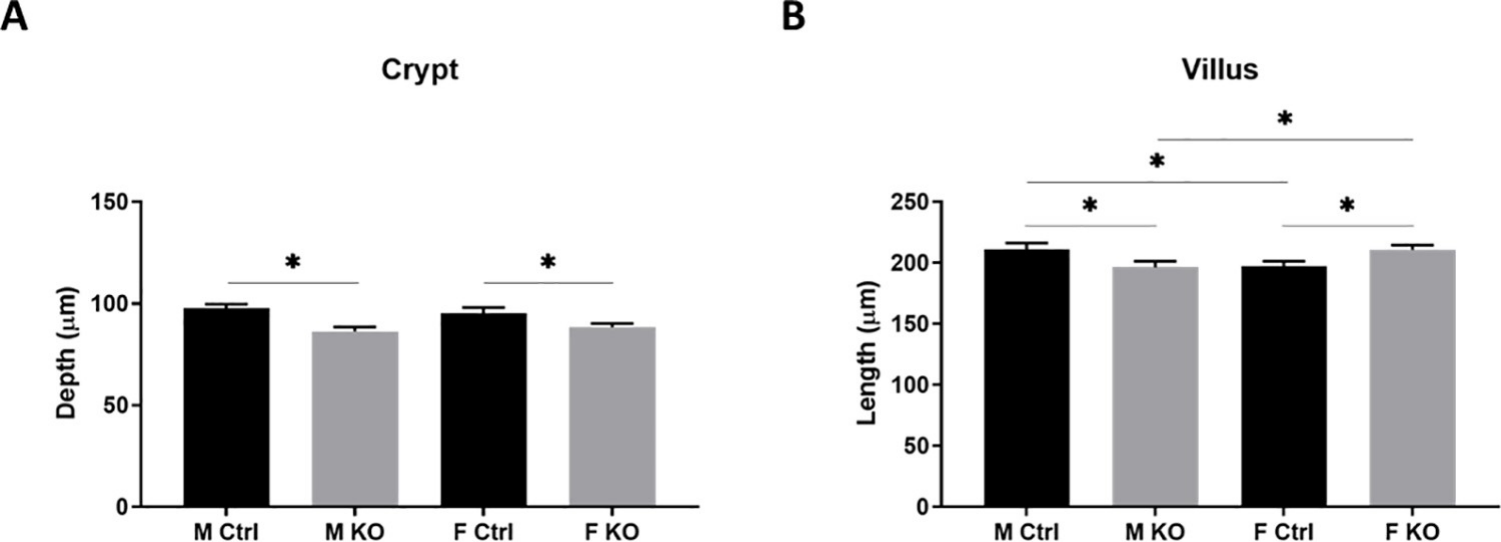
Villi length and crypt depth in VC-Slfn3KO mice. VC-Slfn3KO mice do exhibit (A) villus length and (B) crypt depth that is different from Ctrl mice. (n = 4 mice/group, average of 13–21 villi per sample, average of 14–24 crypts per sample).

Mice carrying a flexible gene-trap knockout-first, *lacZ*, tagged insertion allele of Slfn3 (Slfn3^βgeo^ mice) were created at the UC Davis KOMP Repository (Fig 1). These mice were further bred with B6.129S4-*Gt(ROSA)26Sor*^tm2(FLP*)Sor^/J (Flpo) mice and then B6.Cg-Tg(Vil1-cre)1000Gum/J (VilCre) mice (Fig 1 and S1 Fig). Validation of Vil-Cre, intestinal epithelial cell-specific knock out of Slfn3 was confirmed by single-molecule RNA *in situ* hybridization (Fig 2A). VC-Slfn3KO mice have little to no Slfn3+ intestinal epithelial cells compared to Ctrl mice (Fig 2B). Slfn3^+^ immune cells are seen within the lamina propria of the villi indicating the Vil-Cre specificity to the intestinal epithelial cells. The loss of Slfn3 in pan-Slfn3KO mice caused a decrease in ileal villus length in males, an increase in ileal villus length in female Slfn3KO mice, and a decrease in crypt depth in both male and female Slfn3KO mice [16]. We observed the same significant changes in villus length and crypt depth for the VC-Slfn3KO mice in both the male and female mice (Fig 3).

### Slfn3 influences Slfn family member expression

**Fig 4:**
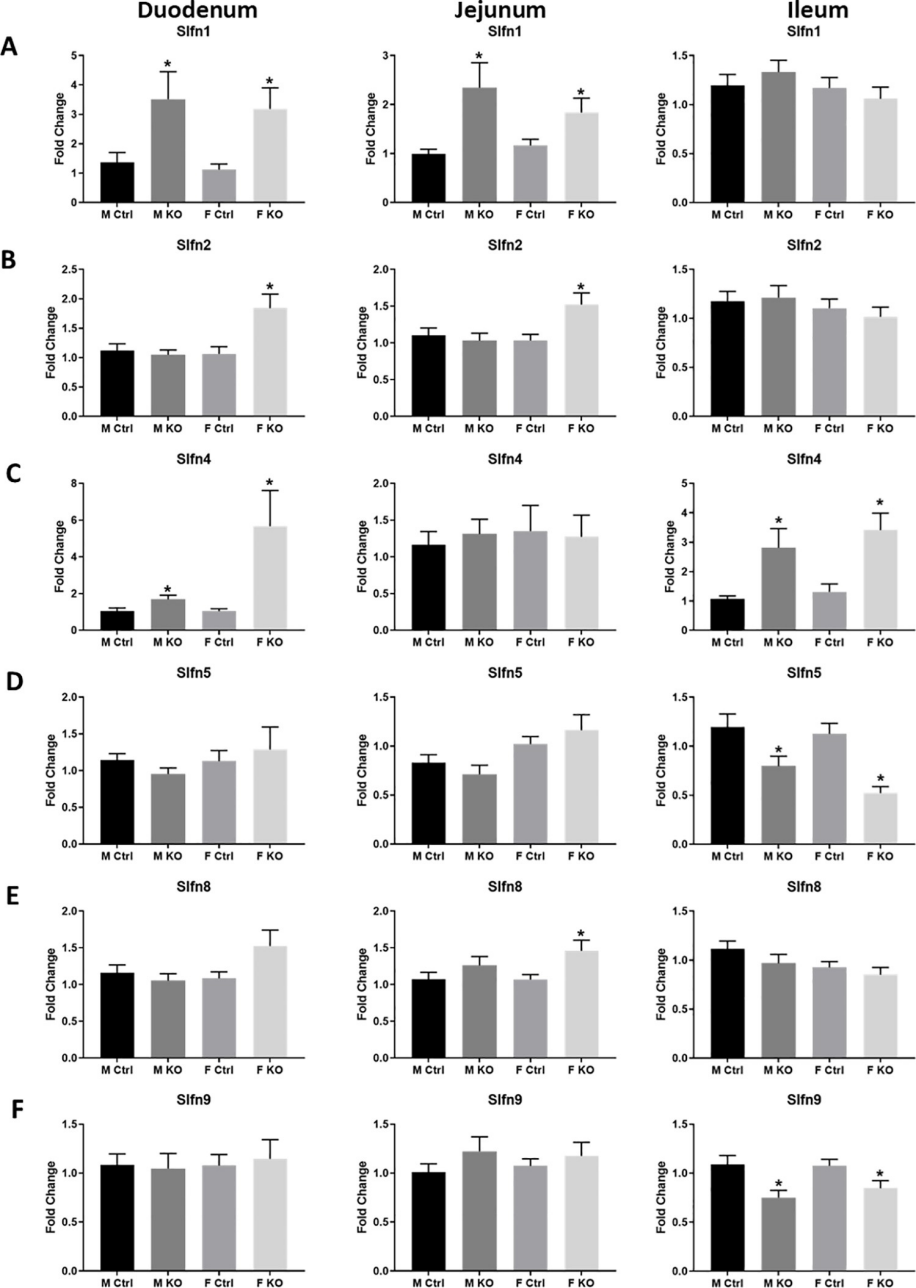
Slfn family expression changes based on intestinal epithelial specific loss of Slfn3, intestinal region, and sex. Total RNA was isolated from intestinal mucosa of the duodenum, jejunum, and ileum of control (Ctrl) and VC-Slfn3KO (KO) mice. The mRNA expression of (A) Slfn1, (B) Slfn2, (C) Slfn4, (D) Slfn5, (E) Slfn8, and (F) Slfn9 was analyzed by qPCR using RPLP0 as a reference control gene. (n = 9–48, *p<0.05 to respective Ctrl).

Previously, we demonstrated that expression of Slfn family members are affected by the loss of Slfn3 in the ileum of pan-Slfn3KO mice [18]. The loss of Slfn3 in the intestinal epithelial cells of VC-Slfn3KO mice also affected the expression of Slfn family members in the duodenal, jejunal, and ileal mucosa. Slfn1 is a part of the Group I Slfn family and was increased in expression in both duodenal and jejunal mucosa of male and female VC-Slfn3KO mice compared to Ctrl mice (Fig 4A). Slfn2, the other murine member of Group I, was increased in expression in female VC-Slfn3KO mice compared to Ctrl mice, but not in male mice (Fig 4B). Neither Slfn1 or 2 had expression differences between VC-Slfn3KO and Ctrl mice in the ileal mucosa (Fig 4B and 4C). Slfn4, a Group II Slfn closely related to Slfn3, was increased in expression in the duodenal and ileal mucosa of both male and female VC-Slfn3KO but did not change in expression in jejunal mucosa in comparison to the Ctrl mice (Fig 4C). Finally, the Group III Slfn members Slfn5 and 9 displayed a decrease in expression in both male and female VC-Slfn3KO mice in the ileal region and Slfn8 only increased significantly in female VC-Slfn3KO jejunum (Fig 4D–4F).

We also evaluated the expression of Slfn family members in the spleen. We did not expect to see a change in Slfn family expression in these VC-Slfn3KO mice due to the Vil-Cre being epithelial-specific for knocking out Slfn3, as we had seen in the pan-Slfn3KO mice [18]. Slfn3 RNA expression was present in all groups (S3 Fig). There was an overall trending decrease in Slfn family expression in the VC-Slfn3KO mice and a significant decrease in Slfn9 in the female VC-Slfn3KO mice in comparison to Ctrl mice (S3 Fig). Slfn4 RNA expression was undetected in both Ctrl and VC-Slfn3KO splenic tissues.

### Differentiation marker expression of SI, Vil1, and Dpp4 are influenced by epithelial Slfn3 loss and by sex

**Fig 5:**
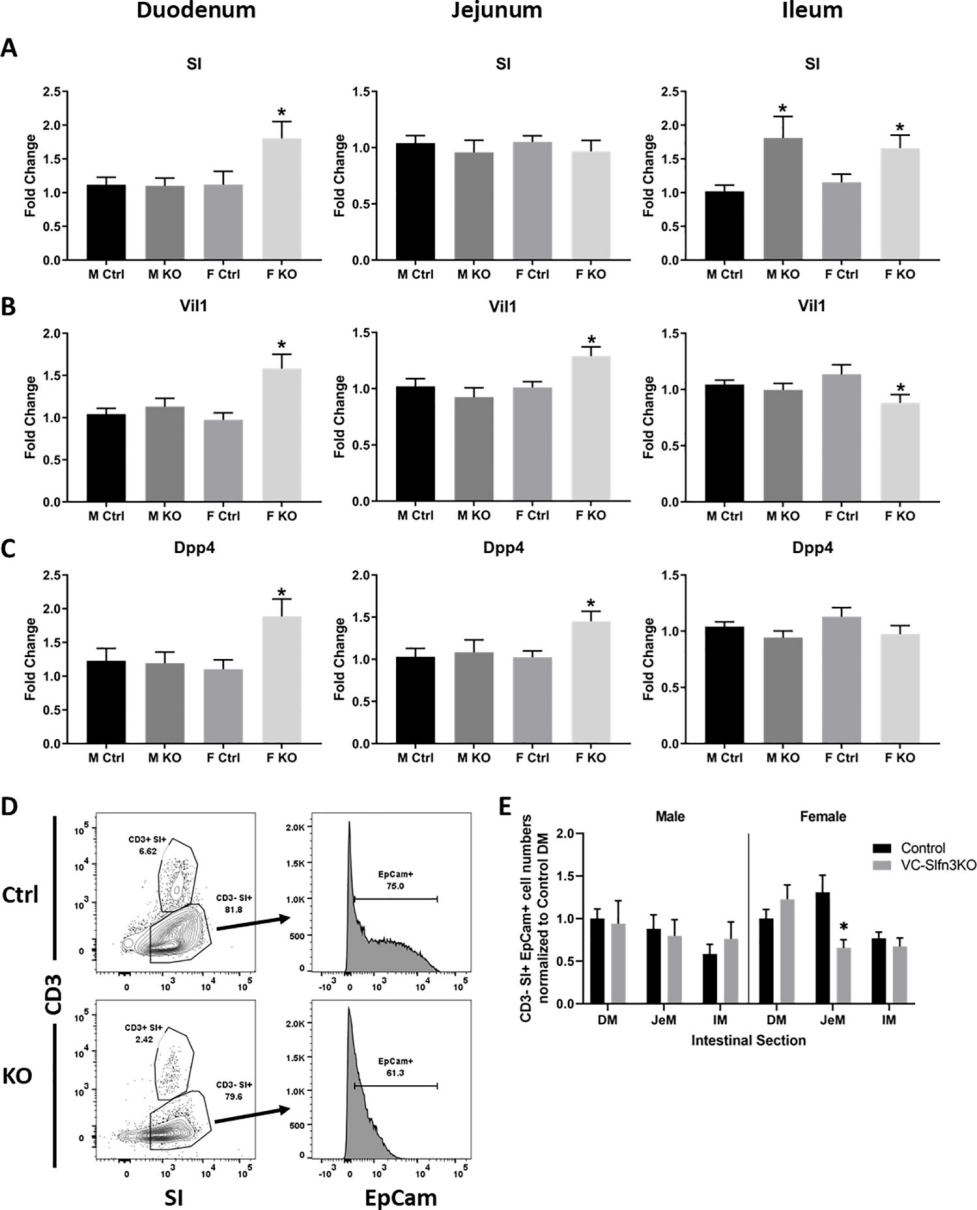
Intestinal differentiation markers SI, Vil1, and Dpp4 vary expression due to intestinal epithelial specific loss of Slfn3, intestinal region, and sex. Total RNA was isolated from intestinal mucosa of the duodenum, jejunum, and ileum of control (Ctrl) and VC-Slfn3KO (KO) mice. The mRNA expression of (A) SI, (B) Vil1, and (C) Dpp4 was analyzed by qPCR using RPLP0 as a reference control gene. (n = 9–50, *p<0.05 to respective Ctrl). Single cell suspension of intestinal cells from the duodenum, jejunum, and ileum were analyzed by flow cytometry in Ctrl and VC-Slfn3KO mice. (D) Representative dot plot gating on CD3^-^ SI^+^ from jejunal intestinal cells were then further gated on EpCam^+^ cells by histogram. (E) CD3^-^ SI^+^ EpCam^+^ cells were normalized to control duodenum cell numbers within each experiment and by sex. (male n = 14–15, female n = 9–14; *p<0.05 to respective Ctrl).

We have chosen to study SI, Vil1, and Dpp4 as markers of intestinal differentiation. SI and Dpp4 are brush border digestive enzymes and have been established as canonical markers for enterocytic differentiation [22–25]. Vil1 is found within the microvillus core of the brush border and is a key calcium regulated actin binding protein [26–29]. In the duodenum, female VC-Slfn3KO mice exhibited increased SI, Vil1, and Dpp4 expression compared to controls (Fig 5A–5C), but male knockouts did not. A similar female-only increase in differentiation markers was observed in the jejunum of the female VC-Slfn3KO except that SI was similarly expressed between male and female Ctrl and VC-Slfn3KO mice. SI was increased in the ileal mucosa of both male and female VC-Slfn3KO mice but surprisingly Vil1 was decreased in the female VC-Slfn3KO mice while Dpp4 did not differ significantly among the groups. Since SI had such a marked change in RNA expression in both male and female VC-Slfn3KO mice, we evaluated SI protein expression by flow cytometry in EpCam^+^ epithelial cells, excluding CD3^+^ immune cells (which also express Slfn3 and SI). SI protein expression changed based on sex and intestinal region. Enterocytes from male VC-Slfn3KO mice did not achieve a statistically significant change in SI protein levels compared to Ctrl mice. However, female VC-Slfn3KO mice had significantly less SI protein in jejunal enterocytes compared to Ctrl mice (Fig 5D and 5E). The CD3^+^ SI^+^ immune cells were evaluated as well, and we found that there was a decrease in SI protein in the duodenum of female VC-Slfn3KO mice (S4 Fig).

### Glucose transporter expressions were impacted by epithelial Slfn3 loss and by sex

**Fig 6:**
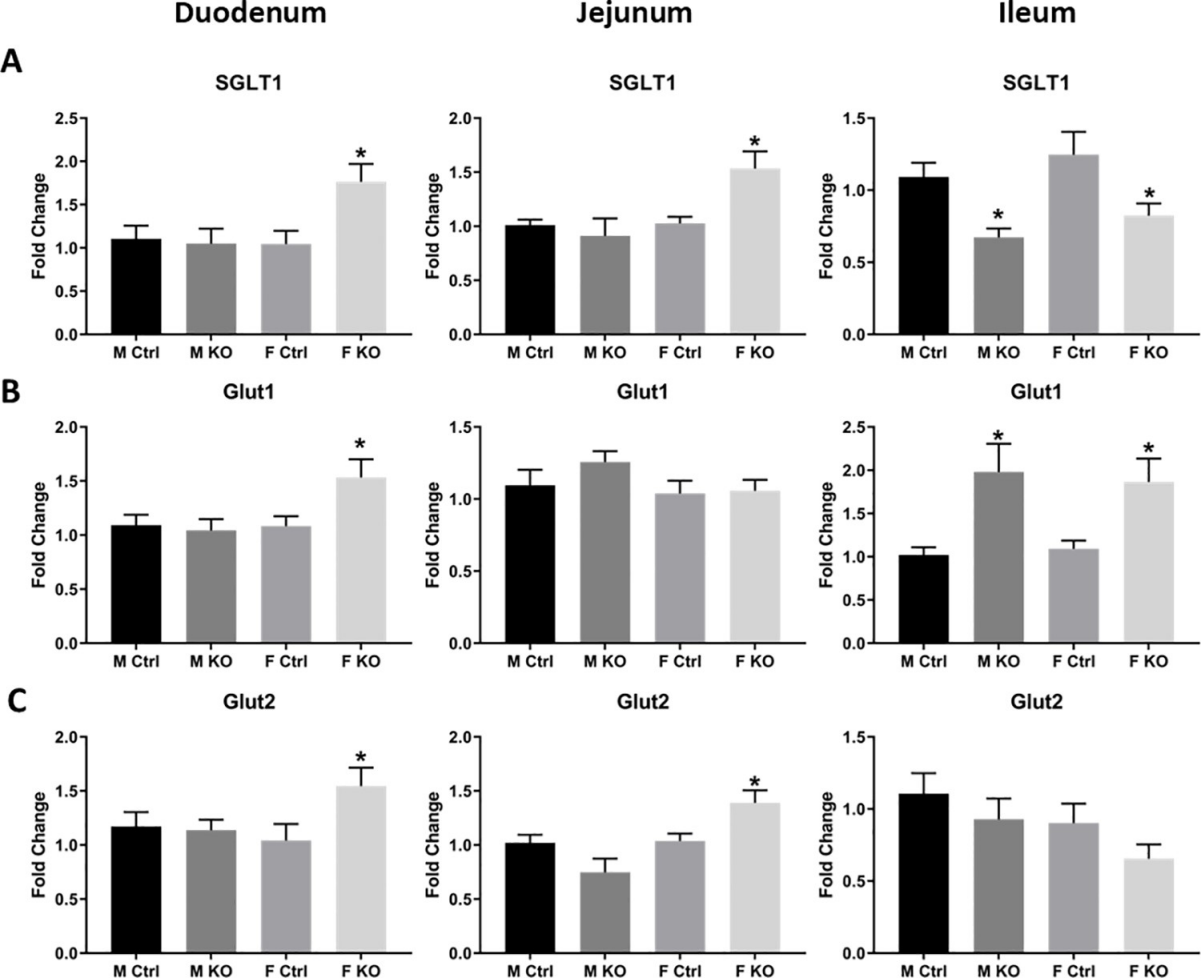
Glucose transporters SGLT1, Glut1, and Glut2 change expression due to intestinal epithelial specific loss of Slfn3, intestinal region, and sex. Total RNA was isolated from intestinal mucosa of the duodenum, jejunum, and ileum of control (Ctrl) and VC-Slfn3KO (KO) mice. The mRNA expression of (A) SGLT1, (B) Glut1, and (C) Glut2 was analyzed by qPCR using RPLP0 as a reference control gene. (n = 9–41, *p<0.05 to respective Ctrl).

Glucose transporters are also important membrane proteins that serve as markers of intestinal epithelial differentiation and functional maturation [11, 30, 31]. Female VC-Slfn3KO mice exhibited increased SGLT1, Glut1, and Glut2 expression in the duodenum and increased SGLT1 and Glut2 in the jejunum, while male mice did not (Fig 6). In the ileum, we observed decreased SGLT1 expression in both male and female VC-Slfn3KO mice, but increased Glut1. Glut2 did not change significantly (Fig 6).

## Discussion

This study investigates the specific role of Slfn3 in intestinal cell differentiation. By creating an epithelial-specific knockout of Slfn3, we were able to investigate the effects of Slfn3 loss in intestinal epithelial cells without possible effects of the loss of Slfn3 in other cell types that could have been observed in the pan-Slfn3KO mouse [16]. We observed that the loss of Slfn3 affected the RNA expression levels of Slfn family members and intestinal differentiation markers based on small intestinal region and sex.

Previously, in pan-Slfn3KO mice, we have shown that the villus length was decreased in male Slfn3KO mice and increased in female Slfn3KO mice in comparison to wild type mice. The crypt depth decreased in both male and female Slfn3KO mice [32]. We observed these same changes to the villus length and crypt depth with the VC-Slfn3KO mice. Additionally, when we transiently interfered with Slfn3 expression in the intestine of male rats by injecting specific siRNA into the lumen of temporarily blocked jejunal segments, villus length was decreased, but there was no change in crypt depth [12]. We and others have published Slfn3 exhibiting antiproliferative properties and that when Slfn3 is lost by siRNA knockdown or increased by ectopic expression or adenovirus, cell proliferation directly correlates [7, 15, 33]. Therefore, these *in vivo* data further indicate a sex-dependent role for Slfn3 in the regulation of villus length and crypt depth.

Previous evaluation of Slfn family member RNA expression in the pan-Slfn3KO mice displayed an increase in Slfn1 and Slfn5 and a decrease in Slfn4, Slfn8, and Slfn9 in the ileal mucosa and was not affected by sex [18]. In this study, we extended our assessment to all intestinal regions and found a sex-dependent increase for Slfn2 and Slfn8 in the duodenal and jejunal regions of the female VC-Slfn3KO vs. Ctrl mice. Ileal Slfn9 decreased in expression for VC-Slfn3KO similarly to what was seen in pan-Slfn3KO. Conversely, ileal Slfn4 expression increased and ileal Slfn5 expression decreased in VC-Slfn3KO, whereas they were oppositely expressed in the pan-Slfn3KO ileum [18] (Table 2). Other studies have shown that Slfn family expression can be induced or reduced due to IFN treatment. Slfn1, 2, 5, and 8 mRNA expression is induced after treatment with IFNα and that knockout mouse embryonic fibroblast cell lines for Stat1 is required for all IFN-inducible Slfn genes, Stat3 is required for all Slfns expect Slfn5, and p38 MAPK is required for Slfn1 and 2 but not Slfn5 and 8 [7, 34]. Additionally, human SLFN5 and SLFN11 are induced by IFN-β, IFN-γ, poly-inosine-cytosine or poly dAdT [35, 36]. We evaluated Slfn family expression in spleen tissue from the VC-Slfn3KO mice. Slfn3 expression was detected in the splenic tissue, which was to be expected in this epithelial-specific KO, however, we did not expect to see a trending decrease in the Slfn family members and a significant decrease in Slfn9 of the VC-Slfn3KO females. A possible explanation could be the fact that *Slfn* paralogues are clustered together and have close sequence similarity, which can lead to potential redundancy in their expressions, functions, and unclear identification of which paralogue is responsible for a particular phenotype [1, 2, 37].

**Table 2:** Comparison of Slfn family and intestinal differentiation marker expression in pan-Slfn3KO mice [16] vs. VC-Slfn3KO mice.

	Pan-Slfn3KO			VC-Slfn3KO		
	Duodenum	Jejunum	Ileum	Duodenum	Jejunum	Ileum
Slfn1	Not tested	Not tested	↑	↑	↑	=
Slfn2			=	F↑	F↑	↓
Slfn4			↓	↑	=	↑
Slfn5			↑	=	=	↓
Slfn8			↓	=	F↑	=
Slfn9			↓	=	=	↓
SI			=	F↑	=	↑
Vil1			=	F↑	F↑	F↓
Dpp4			=	F↑	F↑	=
SGLT1			M↑	F↑	F↑	↓
Glut1			=	F↑	=	↑
Glut2			F↓	F↑	F↑	=

M: male, F: female, =: equal expression, ↑: increased expression, ↓: decreased expression

The brush border enzymes Dpp4 and SI, the actin binding protein Vil1, and the glucose transporters Glut1, Glut2, and SGLT1 are important markers of intestinal differentiation and functional maturation [22–26, 28–30]. The intestinal differentiation markers and glucose transporters displayed a sex-skewed increase in expression for female VC-Slfn3KO mice, except for a decrease in ileal Vil1 and ileal SGLT1 (Table 2). Pan-Slfn3KO mice only exhibited an increase in ileal SGLT1 in males and a decrease in ileal Glut2 in females [32]. Alternatively, in 50% bowel-resected pan-Slfn3KO mice, SI, Dpp4, Glut2, and SGLT1 are all decreased [38]. Additionally, a more correlative effect was seen in temporarily blocked jejunal regions of rats that were injected intraluminally with either adenoviral Slfn3 or Slfn3 siRNA. In these studies, the RNA and protein expression of SI, Vil1, and Glut2 increased with the exposure to Slfn3 adenovirus while suppression of Slfn3 by siRNA led to a decrease in these same markers [12]. In contrast, we found that in the VC-Slfn3KO mice SI RNA expression was increased in the ileum and in the duodenum only for female VC-Slfn3KO mice. But then after protein evaluation, we only found a decrease in SI protein in the jejunum of female VC-Slfn3KO mice. This contrasting SI RNA and protein expression could be similar to how we have previously shown that the human homolog SLFN12, regulates differentiation markers in Caco2 cells and non-malignant HIEC-6 enterocytes via a pathway involving SerpinB12, deubiquitylases UCHL5 and USP14, and the transcription factor Cdx2. Furthermore, Slfn12 regulates ZEB1 and Slug protein conversely to the mRNA in breast cancer cells and c-Myc in lung adenocarcinoma cells [39, 40]. One other possibility could also be that the SI RNA expression was measured from mucosa which includes epithelial and immune cells. Whereas the SI protein expression was measured in only EpCam^+^ epithelial cells and the CD3^+^ immune cells were gated out. However, the protein expression of SI in the CD3^+^ immune cells does not mirror the overall mucosal SI protein levels. Therefore, the differences seen between SI RNA and protein expression could be the combination of expressions between mucosal versus epithelial cells or it could indicate how Slfn3 regulates SI expression at the translational level possibly similarly to SLFN12.

The influence of gender on the expression of intestinal genes and differentiation markers has been less studied in mice and humans but has been most extensively studied in *Drosophila*. A human metabolic syndrome study found that women had decreased plasma Dpp4 activity levels in the metabolic syndrome patients compared to control patients [41]. Renal Glut1 and SGLT1 mRNA expression levels have been shown to be higher in female mice compared to male mice [42]. Interesting work done in *Drosophila* midgut, by Hudry et al. revealed that there are sex differences in intestinal stem cell proliferation that was adult-reversible and intrinsic to the stem cells [43]. They went on to determine that in females, genes associated with cell division-related processes were more greatly expressed, while males had a greater expression of genes coding for proteins that function in carbohydrate metabolism and redox processes [43]. These initial studies suggested that sex-biased intestinal metabolism could contribute to the sex differences observed in whole-body physiology. A subsequent study revealed a bi-directional communication between the male gonad and the adjacent intestinal midgut [44]. This study showed that the testis promotes sex differences in carbohydrate metabolism via a type I family of cytokines called Unpaired (Upd) in *Drosophila*, which are similar to mammalian interleukins and leptin (which are also highly sexually dimorphic in humans and rodents) [44]. The testis-secreted cytokines promoted enterocyte JAK-STAT signaling within the adjacent intestinal midgut leading to sugar gene expression and gut-derived citrate which promoted food intake and sperm maturation [44]. Correlatively, the JAK-STAT pathway is the most important pathway in regulating IFN-inducible genes [34, 45–48]. Therefore, since Slfn expression is induced by IFNs [34], it could be possible that the sex-biased gene expression changes that we observed in this study could be due to an interruption of JAK-STAT signaling with the loss of Slfn3.

## Conclusions

Overall, our targeted deletion of Slfn3 using a Vil-Cre mouse model allowed us to evaluate the effects of Slfn3 loss on Slfn family members and intestinal differentiation markers in intestinal epithelial cells. We not only found that the loss of Slfn3 did influence the expression of Slfn family members and intestinal differentiation markers, but also that this influence varied along the length of the small intestine and was largely sex-biased to females. While the focus of this study was on the small intestine, future investigations may consider how this epithelial Slfn3 knockout affects other epithelia such as gastric, colonic, renal, and pulmonary. Additional future studies could also explore sex-secreted cytokines possibly influenced by the JAK-STAT pathway. Further determining the pathways that Slfn3 is involved in will allow us to focus pathway analysis in the human ortholg, SLFN12. We could then target these pathways for treatment in intestinal diseases, including intestinal diseases that display gender bias, such as inflammatory bowel disease in women.

## Supporting information

S1 Fig Breeding scheme utilized to develop the VC-Slfn3KO mice. The Slfn3^βgeo^ mice were created at the UC Davis KOMP Repository. The Slfn3^βgeo^ mice were breed with B6.129S4-*Gt(ROSA)26Sor*^tm2(FLP*)Sor^/J (Flpo) mice. The resulting Slfn3^flox+/+^Flp^+/+^ pups were backcrossed with C57Bl/6J mice to remove the Flp transgene. These subsequent pups, Slfn3^flox+/-^ were crossed together to generate Slfn3^flox-/-^ mice. The Slfn3^flox-/-^ mice were bred with B6.Cg-Tg(Vil1-cre)1000Gum/J mice. The resultant mice, Slfn3^flox+/-^/ Flp^-/-^/Cre^+/-^, were bred together and then the subsequent Slfn3^flox+/+^/ Flp^-/-^/Cre^+/-^ mice were bred with Slfn3^flox+/+^/Flp^-/-^ to ultimately generate the Slfn3^flox+/+^/ Flp^-/-^/Cre^-/-^ Control (Ctrl) mice and the Slfn3^flox+/+^/ Flp^-/-^/Cre^+/-^ VC-Slfn3KO mice.
(TIF)

S2 Fig Genotyping analysis during the development of VC-Slfn3KO mice. All of the following PCR reactions are described in detail in the Methods section. (A) The genotyping of the Slfn3^βgeo^ mice utilized 3 PCR reactions. A 497bp signal in the WT reaction alone signified wild type (WT) mice. Slfn3^βgeo^ mice had a 362bp signal in the LoxF reaction, a 606bp reaction in the NeoF reaction, and no signal in the WT reaction. Heterozygous (HT) mice had a signal in all 3 PCR reactions. (B) The Slfn3^flox+/-^/Flp^+/+^ mice had a 362bp signal in the LoxF reaction, no signal in the NeoF reaction, and 2 signals of 706bp and 497bp in the WT reaction. Heterozygosity for the Flp transgene was detected from 2 signals of 650bp and 340bp with the Flp reaction. (C) The Slfn3^flox+/+^/ Flp^-/-^/Cre^+/-^ (VC-Slfn3KO) and Slfn3^flox+/+^/ Flp^-/-^/Cre^-/-^ (Control (Ctrl) mice) were genotyped with qPCR melt curve analysis of the LoxF reaction. A signal peak at 81°C indicated Slfn3flox-/- mice, while a signal peak at 78°C indicated Slfn3flox+/+ mice. (D) To determine the presence of the Vil-Cre insert a qPCR melt curve with one signal at 83°C indicated Cre^-^ mice while 2 signals at 78°C and 83°C indicated Cre^+/-^ mice.
(TIF)

S3 Fig Trending decrease in Slfn family RNA expression from the splenic tissue of VC-Slfn3KO mice. Total RNA was isolated from spleen tissue of control (Ctrl) and VC-Slfn3KO (KO) mice. The mRNA expression of Slfn1, Slfn2, Slfn3, Slfn5, Slfn8, and Slfn9 was analyzed by qPCR using RPLP0 or HPRT as a reference control gene. (n = 7–9, *p<0.05 to respective Ctrl).
(TIF)

S4 Fig CD3^+^ immune cells have decreased SI protein in the duodenum of female VC-Slfn3KO mice. Single cell suspension of intestinal cells from the duodenum, jejunum, and ileum were analyzed by flow cytometry in Ctrl and VC-Slfn3KO mice. CD3^+^ SI^+^ EpCam^-^ cells were normalized to control duodenum cell numbers within each experiment and by sex. (male n = 14–15, female n = 9–14; *p<0.05 to respective Ctrl).
(TIF)
