# Supplementary figures and images for: Vil-Cre specific Schlafen 3 knockout mice exhibit sex-specific differences in intestinal differentiation markers and Schlafen family members expression levels
Source: PLoS One. 2021 Oct 28;16(10):e0259195. doi: 10.1371/journal.pone.0259195 (PMC8553116; doi:10.1371/journal.pone.0259195)

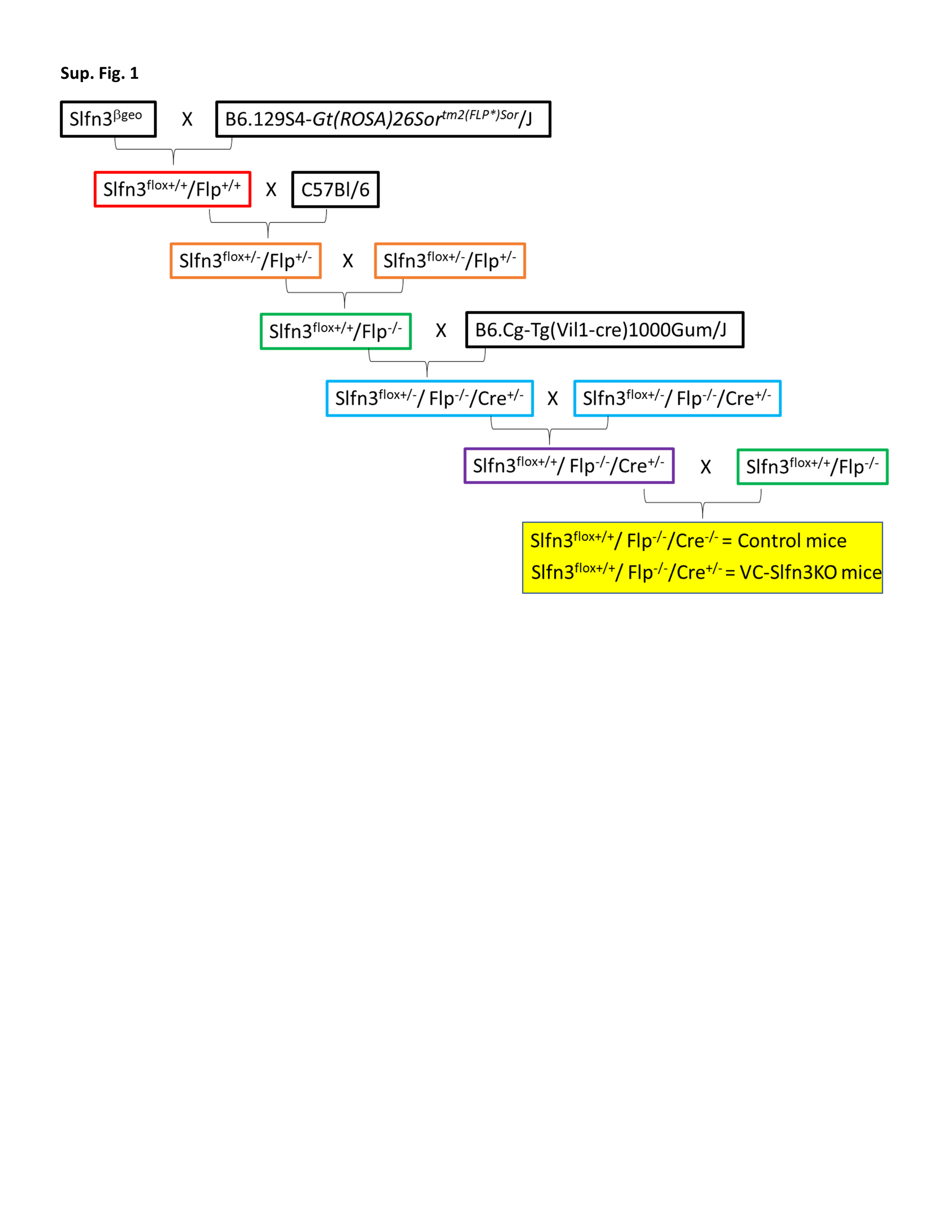

Supplement: S1 Fig — The Slfn3βgeo mice were created at the UC Davis KOMP Repository. The Slfn3βgeo mice were breed with B6.129S4-Gt(ROSA)26Sortm2(FLP*)Sor/J (Flpo) mice. The resulting Slfn3flox+/+Flp+/+ pups were backcrossed with C57Bl/6J mice to remove the Flp transgene. These subsequent pups, Slfn3flox+/- were crossed together to generate Slfn3flox-/- mice. The Slfn3flox-/- mice were bred with B6.Cg-Tg(Vil1-cre)1000Gum/J mice. The resultant mice, Slfn3flox+/-/ Flp-/-/Cre+/-, were bred together and then the subsequent Slfn3flox+/+/ Flp-/-/Cre+/- mice were bred with Slfn3flox+/+/Flp-/- to ultimately generate the Slfn3flox+/+/ Flp-/-/Cre-/- Control (Ctrl) mice and the Slfn3flox+/+/ Flp-/-/Cre+/- VC-Slfn3KO mice. (TIF) [file pone.0259195.s001.tif]

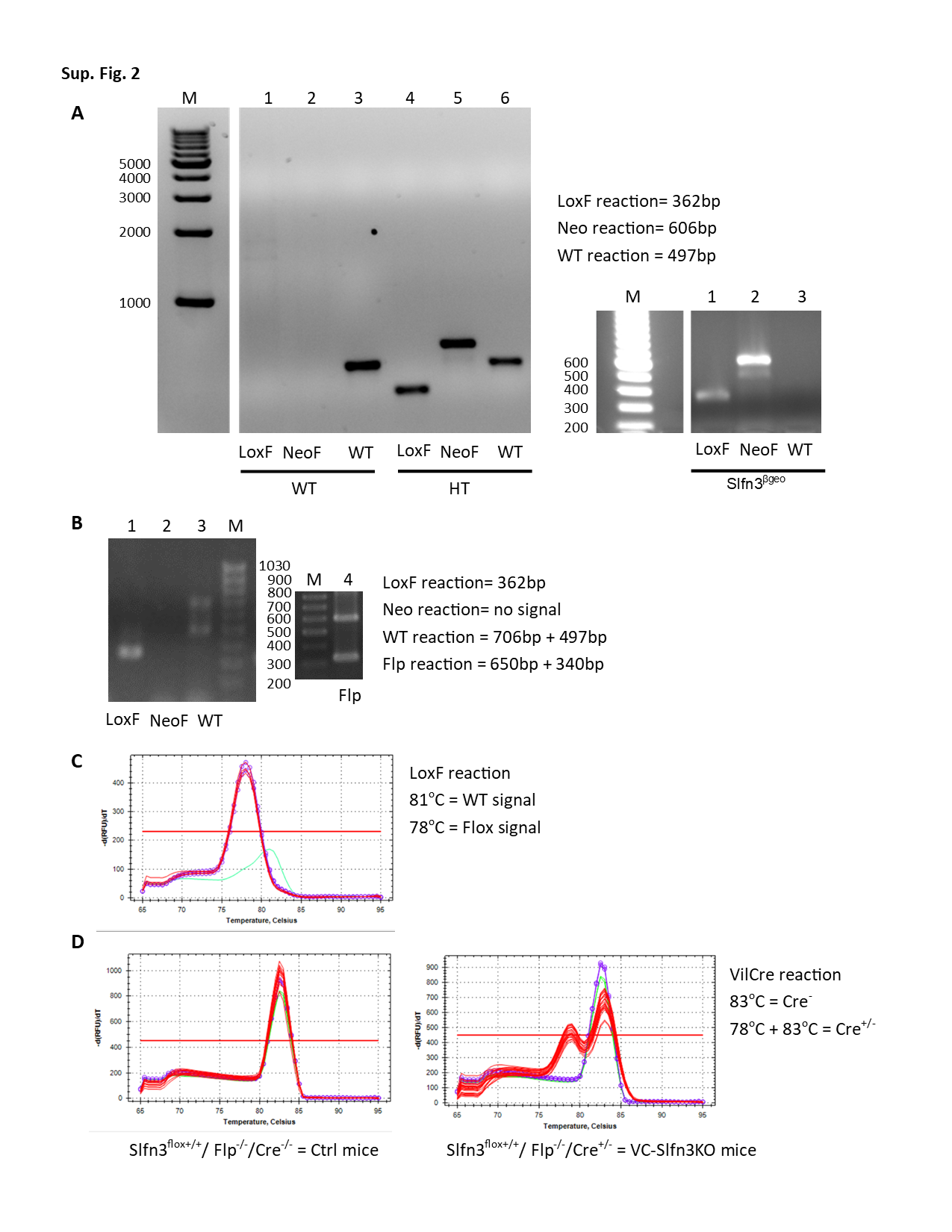

Supplement: S2 Fig — All of the following PCR reactions are described in detail in the Methods section. (A) The genotyping of the Slfn3βgeo mice utilized 3 PCR reactions. A 497bp signal in the WT reaction alone signified wild type (WT) mice. Slfn3βgeo mice had a 362bp signal in the LoxF reaction, a 606bp reaction in the NeoF reaction, and no signal in the WT reaction. Heterozygous (HT) mice had a signal in all 3 PCR reactions. (B) The Slfn3flox+/-/Flp+/+ mice had a 362bp signal in the LoxF reaction, no signal in the NeoF reaction, and 2 signals of 706bp and 497bp in the WT reaction. Heterozygosity for the Flp transgene was detected from 2 signals of 650bp and 340bp with the Flp reaction. (C) The Slfn3flox+/+/ Flp-/-/Cre+/- (VC-Slfn3KO) and Slfn3flox+/+/ Flp-/-/Cre-/- (Control (Ctrl) mice) were genotyped with qPCR melt curve analysis of the LoxF reaction. A signal peak at 81°C indicated Slfn3flox-/- mice, while a signal peak at 78°C indicated Slfn3flox+/+ mice. (D) To determine the presence of the Vil-Cre insert a qPCR melt curve with one signal at 83°C indicated Cre- mice while 2 signals at 78°C and 83°C indicated Cre+/- mice. (TIF) [file pone.0259195.s002.tif]

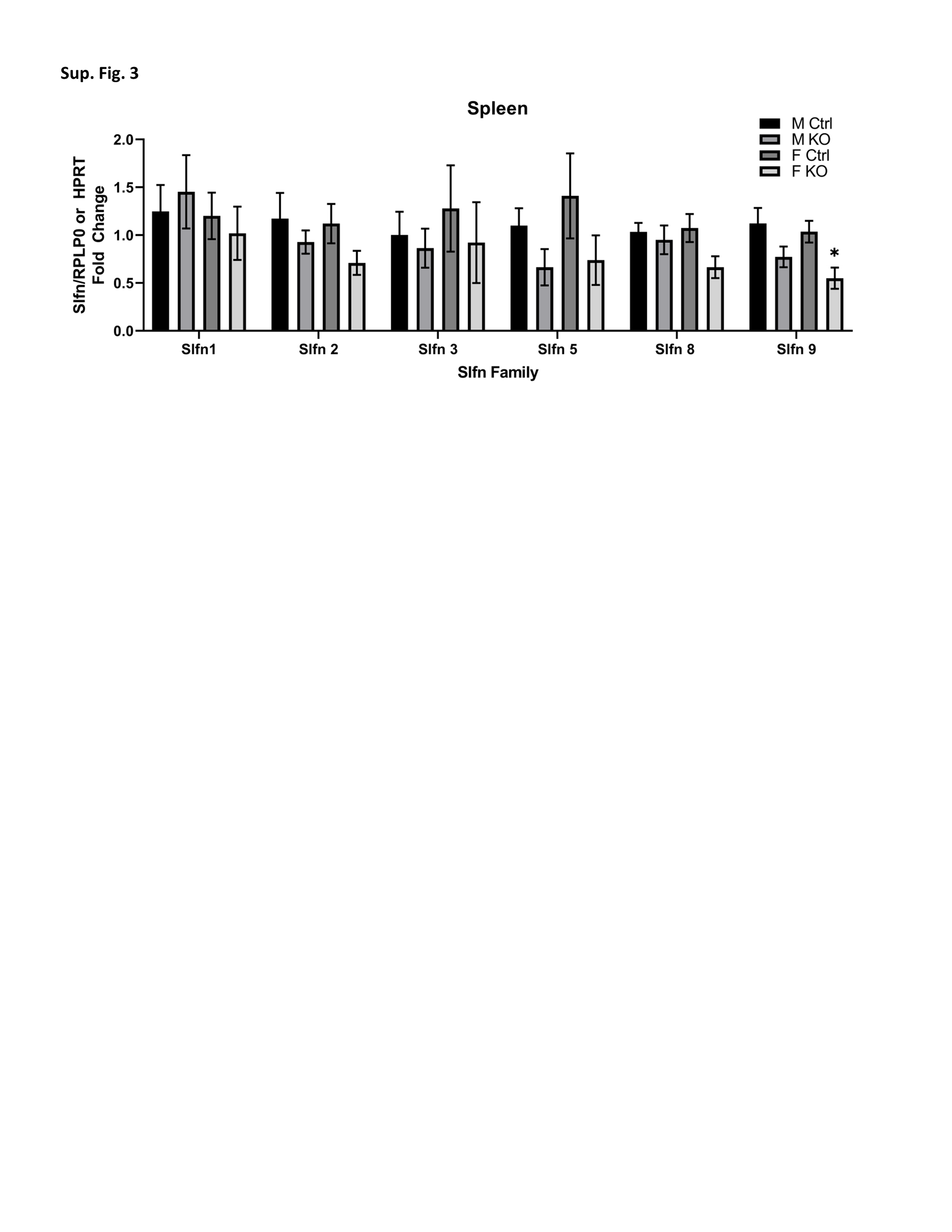

Supplement: S3 Fig — Total RNA was isolated from spleen tissue of control (Ctrl) and VC-Slfn3KO (KO) mice. The mRNA expression of Slfn1, Slfn2, Slfn3, Slfn5, Slfn8, and Slfn9 was analyzed by qPCR using RPLP0 or HPRT as a reference control gene. (n = 7–9, *p<0.05 to respective Ctrl). (TIF) [file pone.0259195.s003.tif]

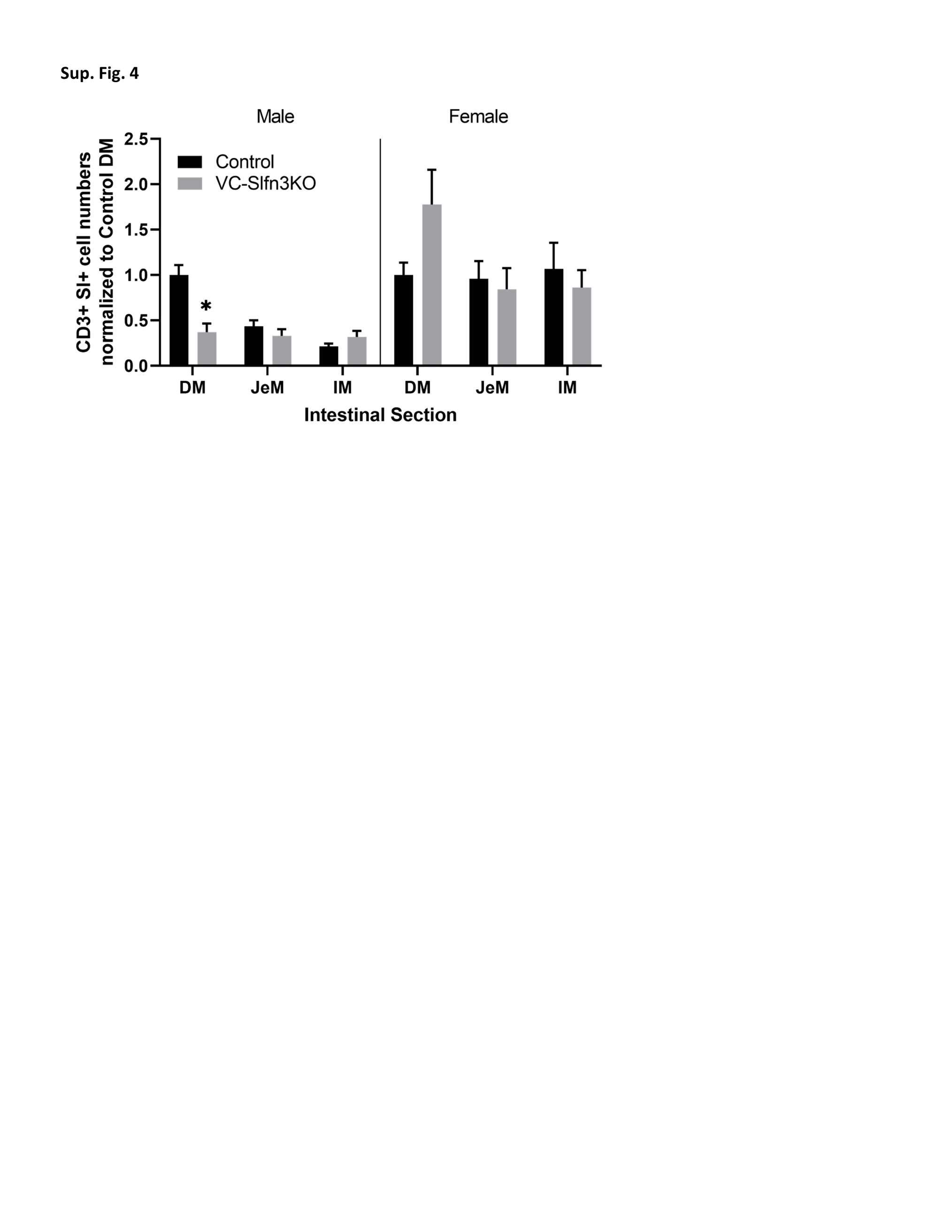

Supplement: S4 Fig — Single cell suspension of intestinal cells from the duodenum, jejunum, and ileum were analyzed by flow cytometry in Ctrl and VC-Slfn3KO mice. CD3+ SI+ EpCam- cells were normalized to control duodenum cell numbers within each experiment and by sex. (male n = 14–15, female n = 9–14; *p<0.05 to respective Ctrl). (TIF) [file pone.0259195.s004.tif]
